# Combination Chemotherapy of Multidrug-resistant Early-stage Colon Cancer: Determining Optimal Dose Schedules by High-performance Computer Simulation

**DOI:** 10.1158/2767-9764.CRC-22-0271

**Published:** 2023-01-09

**Authors:** Chase Cockrell, David E. Axelrod

**Affiliations:** 1 Department of Surgery, University of Vermont College of Medicine, Burlington, Vermont.; 2 Department of Genetics, and Cancer Institute of New Jersey, Rutgers University, Piscataway, New Jersey.

## Abstract

The goal of this project was to utilize mechanistic simulation to demonstrate a methodology that could determine drug combination dose schedules and dose intensities that would be most effective in eliminating multidrug-resistant cancer cells in early-stage colon cancer. An agent-based model of cell dynamics in human colon crypts was calibrated using measurements of human biopsy specimens. Mutant cancer cells were simulated as cells that were resistant to each of two drugs when the drugs were used separately. The drugs, 5-flurouracil and sulindac, have different mechanisms of action. An artificial neural network was used to generate nearly 200,000 two-drug dose schedules. A high-performance computer simulated each dose schedule as a *in silico* clinical trial and evaluated each dose schedule for its efficiency to cure (eliminate) multidrug-resistant cancer cells and its toxicity to the host, as indicated by continued crypt function. Among the dose schedules that were generated, 2,430 dose schedules were found to cure all multidrug-resistant mutants in each of the 50 simulated trials and retained colon crypt function. One dose schedule was optimal; it eliminated multidrug-resistant cancer cells with the minimum toxicity and had a time schedule that would be practical for implementation in the clinic. These results demonstrate a procedure to identify which combination drug dose schedules could be most effective in eliminating drug-resistant cancer cells. This was accomplished using a calibrated agent–based model of a human tissue, and a high-performance computer simulation of clinical trials.

**Significance:**

The results of computer-simulated clinical trials suggest a practical dose schedule for two drugs, 5-fluorouracil and sulindac, that could eliminate multidrug resistant early-stage colon cancer cells with minimum toxicity to the host.

## Introduction

The purpose of cancer therapy is to eliminate existing malignant cells or to intercept premalignant cells from progressing to malignancy. Three modes of therapy include stopping abnormal cells from proliferating (cytotoxic chemotherapy), inhibiting the function of specific proteins (precision therapy), or restoring immune function (immunotherapy). The effective implementation of each of these modes of therapy requires choices, including which drug or combination of drugs to employ, the intensity of the drug (dose), the time during which the drug is applied (duration), and how often the drug is applied (interval). Each of these choices needs to be reevaluated when drug-resistant cancer cells evolve, a frequent clinical concern (1).

The purpose of this project was to develop a methodology for determining the optimum drug dose schedules for cancer chemotherapy. This includes optimizing drug combination chemotherapy for multidrug-resistant cancer cells. The challenge was to determine the dose intensity, duration, and interval for each drug that would eliminate multidrug-resistant cancer cells with the least accumulated toxicity. As an example, we focused on colon cancer which starts with the abnormal proliferation of cells in the colon crypt, and two drugs 5-flurouracil (5-FU) and sulindac with distinct mechanisms of action. 5-FU interferes with DNA synthesis and is cytotoxic to dividing cells (2, 3), whereas sulindac induces apoptosis of cells at the crypt lumen interface (4).

In colon cancer, adjuvant chemotherapy is commonly administered after cancer surgery. However, adjuvant chemotherapy has only a modest effect on the 5-year recurrence-free survival of colorectal cancer (5–9). Some recurrence is associated with the evolution of mutant tumor cells that are resistant to one or more chemotherapeutic drugs (10). There are several mechanisms by which cancer cells acquire resistance to chemotherapeutic drugs (11–13). For instance, cancer cells can become resistant to a single drug, such as the widely used 5-FU, through mutation or amplification of the gene coding for the target enzyme thymidylate synthetase (2, 3). Other mechanisms of resistance to 5-FU include enhanced drug efflux due to changes in the ATP-binding cassette protein (14, 15), or a reduction in the rate of cellular division in cancer stem cells (16), dormant cells (17–20), senescent cells (21), persister cells (22, 23), and polyploid cells (24).

Several strategies have been developed to overcome drug resistance of cancer cells. Higher doses of a drug have been suggested (25), but may not be tolerated because of increased host toxicity (26). DeVita and colleagues pioneered the use of drug combinations to overcome resistance to a single drug (27, 28), which has continued to be employed (29). Another strategy to develop modified dose schedules that would be more effective against cancer cells while sparing normal host cells of toxic side effects. Such modified dose schedules include repeated low doses (metronomic therapy), alternating schedules, sequential schedules, and intermittent schedules (30). For instance, Chandra and colleagues (31) used intermittent doses of sulindac for prevention and Leder and colleagues (32) investigated intermittent doses of radiation for the treatment of glioblastoma.

We have previously investigated intermittent dose schedules for the prevention and treatment of colon cancer. An agent-based computer model of cell proliferation in the colon crypt was developed and calibrated using measurements of human biopsy specimens (33). The behavior of the model was verified by reproducing three features of biological crypts that had previously been observed, that is, induction of adenomas by mutations that occurred at the top or bottom of the crypts (34), monoclonal conversion by neutral drift (35), and robust recovery from perturbation by exposure to a cytotoxic agent (36).

We previously used the computer model to determine the optimum dose schedules for the prevention of colon cancer (37, 38) and optimum dose schedules for colon cancer therapy using a single drug (39, 40). However, an optimum dose schedule for two drugs needs to specify the dose intensity, dose duration, and interval of each of the two drugs to accomplish the following objectives: it cures (kills and eliminates) the highest percentage of cancer cells, induces the lowest amount of damage due to drug cytotoxicity, allows crypts to recover and retain function, and has clinically practical time schedules. The goal of this project was to demonstrate a methodology to determine the optimum dose schedules for a combination of two chemotherapeutic drugs that could eliminate multidrug-resistant cancer cells with minimal toxicity to the host. This is a challenging multiobjective optimization problem (41).

Here we report the optimum dose schedules for drug combination therapy in multidrug-resistant early-stage colon cancer. The example drugs 5-FU and sulindac have different mechanisms of action. A high-performance computer was used to simulate hundreds of thousands of clinical trials using different dose schedules. Among these, a subset of dose schedules was determined to eliminate doubly-resistant cancer cells from the colon crypt while retaining crypt function. Notably, we were able to identify specific dose schedules that eliminate cancer cells with the least cytotoxicity, allow recovery of normal crypt function, and have a clinically practical therapy time schedule.

## Materials and Methods

### Colon Crypt Computer Model

Colon_Crypt_Model_041321.nlogo is an agent-based model of cell dynamics in crypts of the human colon. It was revised with new features from previous models, as described in detail (33, 42). This model is available as a Supplementary file. It was developed in the application NetLogo version 5.3.1, and was revised to run in NetLogo version 6.2.0. It does not run on the web version of NetLogo. NetLogo is a multiagent programmable modeling environment. It was authored by Uri Wilenski and developed at the Center for Connected Learning and Computer-Based Modeling. It is a multiplatform (Mac, Windows, or Linux) open-source application. NetLogo version 6.2.0, can be downloaded from http://ccl.northwestern.edu/netlogo/download.shtml.

The model describes cell dynamics in colon crypts, including stem cells, proliferating cells, and differentiated cells. The number of cells in crypts is maintained as new cells are produced from proliferating cells and differentiated cells are removed from the top lumen surface (35).

The model was calibrated with cell numbers measured in a human biopsy specimen (33). A histologic slide containing sections from a biopsy of the sigmoid colon of a normal patient was stained with the anti-body MIB-1 to the proliferating antigen Ki-67, and counterstained with hematoxylin. Positively stained cells were considered proliferating cells. Unstained cells at the bottom of the crypt were considered quiescent stem cells. Unstained cells near the top of the crypt were considered differentiated cells.

The model assumes that the probability that a cell will divide is determined, in part, by its position in a microenvironment gradient along the crypt axis, with the probability of division higher at the bottom than at the top. The probability that a cell will die is determined, in part, by its position in another microenvironment gradient along the crypt axis, with the probability of dying higher at the top than at the bottom. Stem cells at the bottom of the crypt are in a quiescent niche.

Model parameter values of the divide gradient and die gradient were determined that reproduced the mean and variance of each cell type measured in multiple crypts (33). The “Info” tab accessible at the interface of the model contains additional descriptions of each feature of the model, and the basis for choosing each default parameter. The “Code” tab accessible at the interface of the model contains the Netlogo code and extensive comments.

The behavior of the model was verified as reproducing three features of the biological crypts that had previously been observed, that is, induction of adenomas by mutations occurring at the top or at the bottom of the crypts (34), monoclonal conversion by neutral drift (35), and robust recovery from perturbation by exposure to a cytotoxic agent (36).

### High-performance Computer Simulation

To explore the parameter space of chemotherapeutic dose schedules efficiently, the NetLogo crypt model described above was ported to C++ using established methods (43, 44). The C++ files are available as Supplementary files.

The dose schedules for a single drug are defined by three variables: duration, interval, and dose (represented as lethality for cytotoxic drugs and PTreatment for apoptosis-inducing drugs). The duration of the dose is the time during which cells in a crypt are exposed to either a cytotoxic or an apoptotic drug. The interval between the doses was defined as the time from the beginning of one dose to the beginning of the next dose. The cytotoxic treatment computer model parameter, lethality is a factor that increases the probability that a cell will die above that determined by the cell's position in the crypt. The lethality factor affects both normal cells and mutant cells. Apoptotic treatment changes the size of the crypt by altering the probability that a cell at the top of the crypt will die. This is controlled by the computer model parameter PTreatment (CptDieMax). Smaller crypts increase the probability that a cell, normal or cancer, will be removed at the top of the crypt.

Each cytotoxic treatment parameter set consisted of a dose schedule described by a parameter set of one value for duration, one value for interval, and one value of cytotoxic lethality. Each apoptotic treatment parameter set consisted of a dose schedule described by a parameter set of one value of duration, one value of interval, and one value of PTreatment (CptDieMax). For cytotoxic therapy, the duration of treatment was varied from 1 to 48 timesteps with increments of 1 step, the interval of treatment was varied from 2 to 96 steps with increments of 2 steps (with the requirement that the interval is greater than the duration). Each computer step was estimated to correspond to approximately 4 hours of human time based upon the kinetics of recovery from chemotherapy of the model and of humans (45). The lethality factor was varied from 1 to 20 in increments of 0.25. For apoptotic therapy, the duration of treatment was varied from 1 to 48 timesteps with increments of 1 step, the interval of treatment was varied from 2 to 96 steps with increments of 2 steps, and the apoptotic treatment parameter PTreatment (CptDieMax) was varied from 1 to 20 in increments of 0.25. The limit of apoptotic treatment was such that the size of the crypt did not decrease to less than 1,399 total cells, the lowest number measured in biopsy specimens (33).

Each combination of cytotoxic treatment and apoptotic treatment parameter set had a cytotoxic duration, cytotoxic interval, cytotoxic lethality, in combination with apoptotic duration, apoptotic interval, and apoptotic PTreatment (CptDieMax). A comprehensive and/or brute-force exploration of this entire parameter space would require the execution of more than 100 billion simulation runs, rendering the problem computationally intractable to available resources because each set of 50 stochastic replicate simulations takes approximately 2 minutes on a single processing core. To address this challenge, we utilized a previously validated active learning approach (46) to intelligently explore the parameter space, and automatically remove dose schedules that would generate excess toxicity from consideration. In this approach, we began by generating a complete set of combination drug dose schedules, and trained an artificial neural network (ANN) to determine toxicity (whether or not the toxicity of a specific dose schedule would lead to extinguishment of the colon crypt) of a combination drug dose schedule using 100,000 randomly selected combination dose schedules. The trained ANN then makes a toxicity prediction (will this dose schedule be sufficiently toxic to extinguish the crypt) for every enumerated dose schedule; this is tractable as thousands of dose schedule can be evaluated per second compared with the minutes needed to execute 50 stochastic replicates of the simulation. The algorithm then selects the 10,000 dose schedules for which the toxicity classification generated by the current ANN was most uncertain [i.e., the probability of being in class 1 (toxicity below maximum toxicity threshold) and class 2 (dose schedule generates toxicity at or above the maximum toxicity threshold) was closest to 0.5]. The algorithm then runs the simulation using those dose schedules, adds the results to the training set, and retrains the network. This process was continued iteratively until both the sensitivity and specificity of the trained network exceeded 99% (99.6% sensitivity and 99.4% specificity). Ultimately, this required 184,320 combination dose schedules. We then executed the simulations for the dose schedules that had been classified as “nontoxic,” meaning that while colon crypts may have experienced some decrease in size from the chemotherapeutic agent, it would not be extinguished.

The result is that we only had to execute the simulation for 184,320 dose schedules instead of 100 billion. The drawback is that the results are not perfect; there are a small number of false positives and negatives. False positives (i.e., a dose schedule classified as nontoxic when it is actually toxic) are not a problem, as they are discovered when simulating the filtered set of combination dose schedules. False negatives could be potentially problematic as they remain unexplored; however, because such a large parameter space of drug combinations was explored, there were numerous candidate dose schedules that generated low toxicity and excellent cure rates; therefore perfection was not required. Thus, while the results are not perfect, they are justified by the significant computational cost-savings provided by the algorithm.

We recognize that the variability of the simulation-generated toxicity is dependent and interlinked with the mutation rate of crypt cells and the rate at which they are eliminated by the therapy and/or dose schedule. Thus, some of the determined dose schedules would likely be eliminated should they be tested against more stochastic replicates. We have addressed this limitation in two ways: (i) we have subselected clinically tractable dose schedules, and (ii) those dose schedules are not at the boundaries of plausibility. The latter consideration is most important as those dose schedules closest to the boundaries of plausibility will be most sensitive to the increase in stochastic replicates. As illustration, consider a hypothetical dose schedule that has a 100% cure rate with a drug concentration of 7.5 and above, and 100% nontoxic rate with a drug concentration below 15 when measured using 50 stochastic replicates. If one were to double the amount of replicates, one may expect that the cure rate of drug concentrations of 7.5 may dip below 100%, while the number of people who were injured or killed by drug toxicity when given a dose schedule with a drug concentration of 15 may rise above 0. Thus, the most robust dose schedules will be those that meet our filtering criteria (cure all cancer, kill no one due to toxicity) that are located centrally in the dose-schedule parameter space rather than at the boundaries.

### Model Implementation Details

Cancer cells grow faster than normal cells; controlling for their position in the crypt, a cancer cell has a 16% greater probability of dividing and a 10% greater probability of dying than a normal cells. Drug-resistant cancer cells grow faster than normal cells but grow slower than cancer cells. They have an 8% greater probability of dividing than normal cells, and a 5% greater probability of dying than normal cells (37).

Cancer cells or drug-resistant cancer cells were simulated by mutating proliferating cells. Mutations were initiated at 200 steps from the start of each run. Therapy was initiated when cancer cells or drug resistant cancer cells reached 50% of the total number of cells in the crypts. For combination therapy, the cytotoxic treatment and apoptotic treatments were initiated simultaneously. To account for the effects of randomness in the model, 50 stochastic replicates were performed for each dose schedule.

### Data Availability

The computer model codes and data generated in this study are described below. They are available as Supplementary files.

Model: The Netlogo model “Colon_Crypt_Model_041321.nlogo” contains a graphical user interface, explanatory information text, annotated code, and the Behavior Space Tool used to run the code. It is available as a Supplementary file at https://doi.org/10.7282/00000169. The C++ code version of the model is available as four Supplementary files: "agents_axelrodCC.h", "axelrod_cc_simulationFunctions.cpp", "Parameters.h", and "SingleCrypt_ACC.cpp". They are available at https://doi.org/10.7282/00000172.

Data: The 913 different dose schedules that cured all cancer cells in 100% of 50 simulation runs while retaining crypt function are available in the Supplementary Table S1. The 2430 dose schedules that cured all doubly-resistant mutants in 100% of 50 independent trials and allowed crypts to recover are available in the Supplementary Table S2.

## Results

### Specific Dose Schedules of a Cytotoxic Drug can Eliminate Cancer Cells from Colon Crypts While Retaining Crypt Function

Colon cancer begins with the abnormal proliferation of some cells in the normal colon crypt. In normal crypts, the total number of cells is maintained by a balance of cell division among the stem cells and proliferating cells at the bottom two-thirds of the crypt, and new cells moving up the crypt where they are removed by apoptosis at the top of the crypt (35). Abnormal cells, with higher rates of cell division and cell death, may be initiated at the bottom, the lower third, or the top third of the crypt (33, 47, 48). These cells move up the crypt and are removed, but if the population of these cells multiples faster than they can be removed they will fill the crypt and form an adenoma, an early stage in invasive cancer (49). In this project, we refer to these abnormally proliferating, incipient cancer cells simply as cancer cells.

Cytotoxic chemotherapy, with 5-FU which inhibits DNA synthesis, kills dividing cells, both normal and cancer cells. The rationale for using a cytotoxic drug is that rapidly dividing cancer cells are killed more efficiently than slower dividing normal cells and quiescent stem cells. An effective therapeutic index can be achieved in early-stage colon cancer only if a drug dose schedule can be identified to remove cancer cells and maintain crypt function. This can be accomplished using intermittent doses. Between doses, drug-resistant quiescent cells sense a decrease in the number of normal cells, awake from quiescence, actively divide, and a restore the normal number of crypt cells. Thus, the crypt function can be maintained.

We identified intermittent dose schedules for a cytotoxic drug that can eliminate cancer cells from the colon crypt while retaining crypt function. As described in the Materials and Methods section, a high-performance computer was used to generate 184,320 parameter sets, each with a different duration of drug exposure, interval between drug exposure, and lethality. Among these 184,320 parameter sets, 913 different dose schedules cured all cancer cells in 100% of 50 simulation runs, while retaining crypt function (Supplementary Table S1).

**FIGURE 1:**
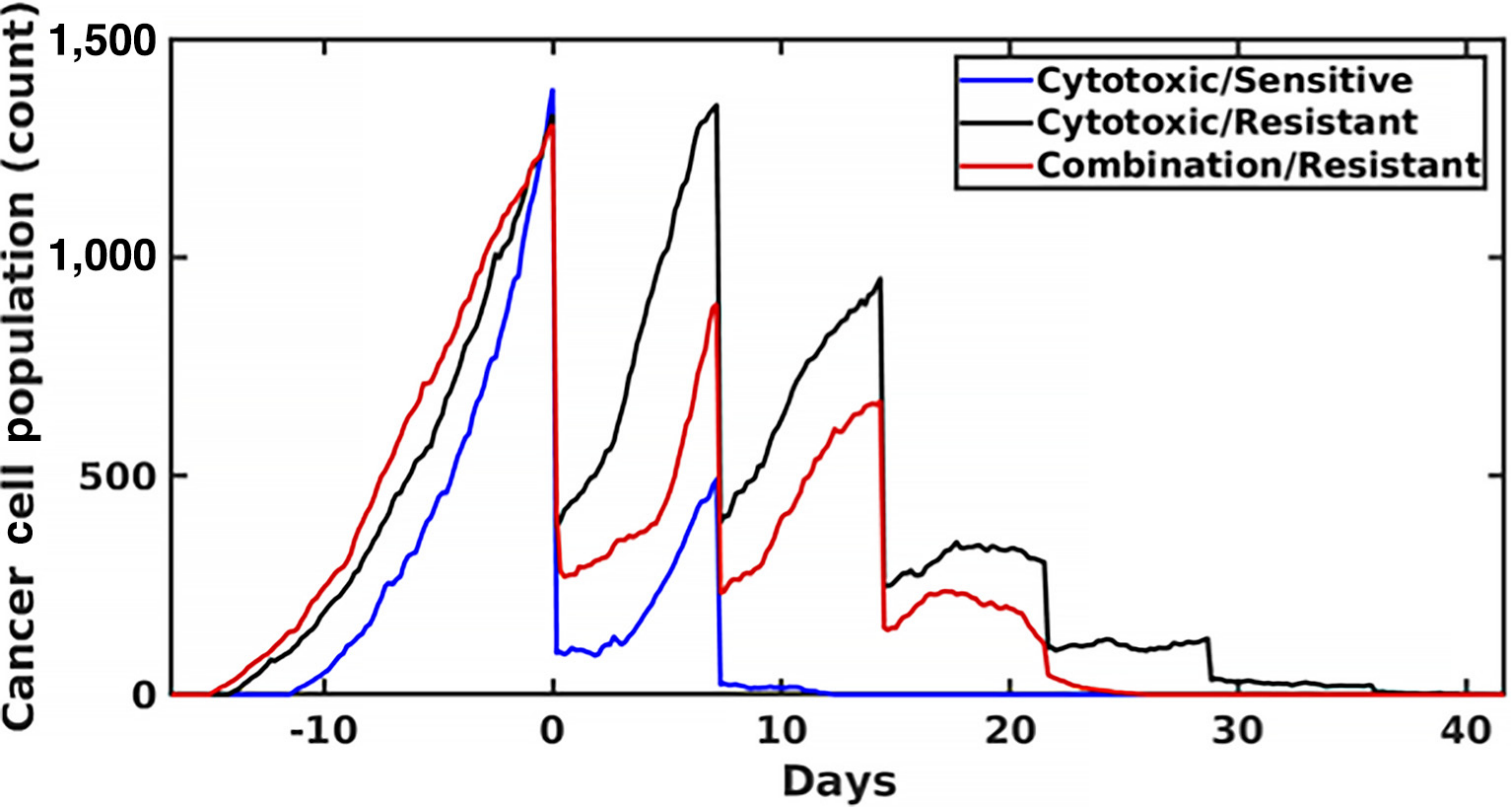
Kinetics of curing cells treated with cytotoxic or combination therapy. The time to cure the sensitive cancer cells treated with cytotoxic treatment is 11.84 days (blue line), the resistant mutant treated with cytotoxic treatment is 32.43 days (black line), and the resistant mutant treated with combination of cytotoxic and apoptotic treatment is 22.17 days (red line).

Among the dose schedules that cured all cancer cells, we selected those that showed the least negative effect on crypt function. An intermittent dose schedule with a duration of 1 step, interval between doses of 42 steps, and lethality of 5.75 reduced the crypt size to 59.27% of the average size of an untreated crypt (Table 1, line 1) and allowed crypts to recover between doses. Cancer cells exposed to this intermittent cytotoxic treatment decreased in number to zero in an average of 71.01 steps (Table 1, line 1), the equivalent to 11.8 days (Fig. 1, Cytotoxic, Sensitive).

**TABLE 1:** Dose schedules and results

		Dose schedule		Percent cure	Time to cure		Crypt size
Cancer cell type (drug response)	Drug treatment	Cytotoxic	Apoptotic	(*n* = 50 runs)	Steps	Days	(% untreated)
1. Sensitive	Cytotoxic	1, 42, 5.75^a^		100	71.01^b^	11.84	59.27^c^
2. Resistant mutant^d^	Cytotoxic	1, 42, 8.25		100	194.56	32.43	63.94
3. Resistant mutant	Apoptotic		1, 84, 3.5	0	N/A	N/A	N/A
4. Resistant mutant	Combination	1, 42, 7.5	1, 84, 3.5	100	133	22.17	48.3
5. Resistant mutant	Cytotoxic	1, 42, 7.5		66	311	51.28	63.01

^a^The numbers, in order, are as follows: duration of dose steps, interval steps between doses, intensity of cytotoxic treatment or of apoptotic treatment (defined in Materials and Methods section).

^b^One step is estimated to be 4 hours.

^c^Indicates the extent of perturbation, crypts recover.

^d^Resistant mutant means relatively resistant compared with sensitive cancer cell, requiring higher cytotoxic dose and longer time to cure.

### Cancer Cells may Evolve Resistance to Multiple Drugs

Mutant cancer cells may evolve to become resistant to a cytotoxic drug (11, 13), including 5-FU in colon cancer (3, 12). Drug-resistant cancer cells in the crypt were modeled as dividing at a slower rate and dying at a lower rate than the drug-sensitive cancer cells described above. In the absence of a cytotoxic drug, the drug-sensitive cancer cells had a division rate of 1.16 ×, and a cell death rate of 1.1 × of normal cells at the same position in the colon crypt; whereas the cancer cells that were relatively drug resistant had a division rate of 1.08 ×, and death rate of 1.05 × of normal cells at the same position in the colon crypt.

**FIGURE 2:**
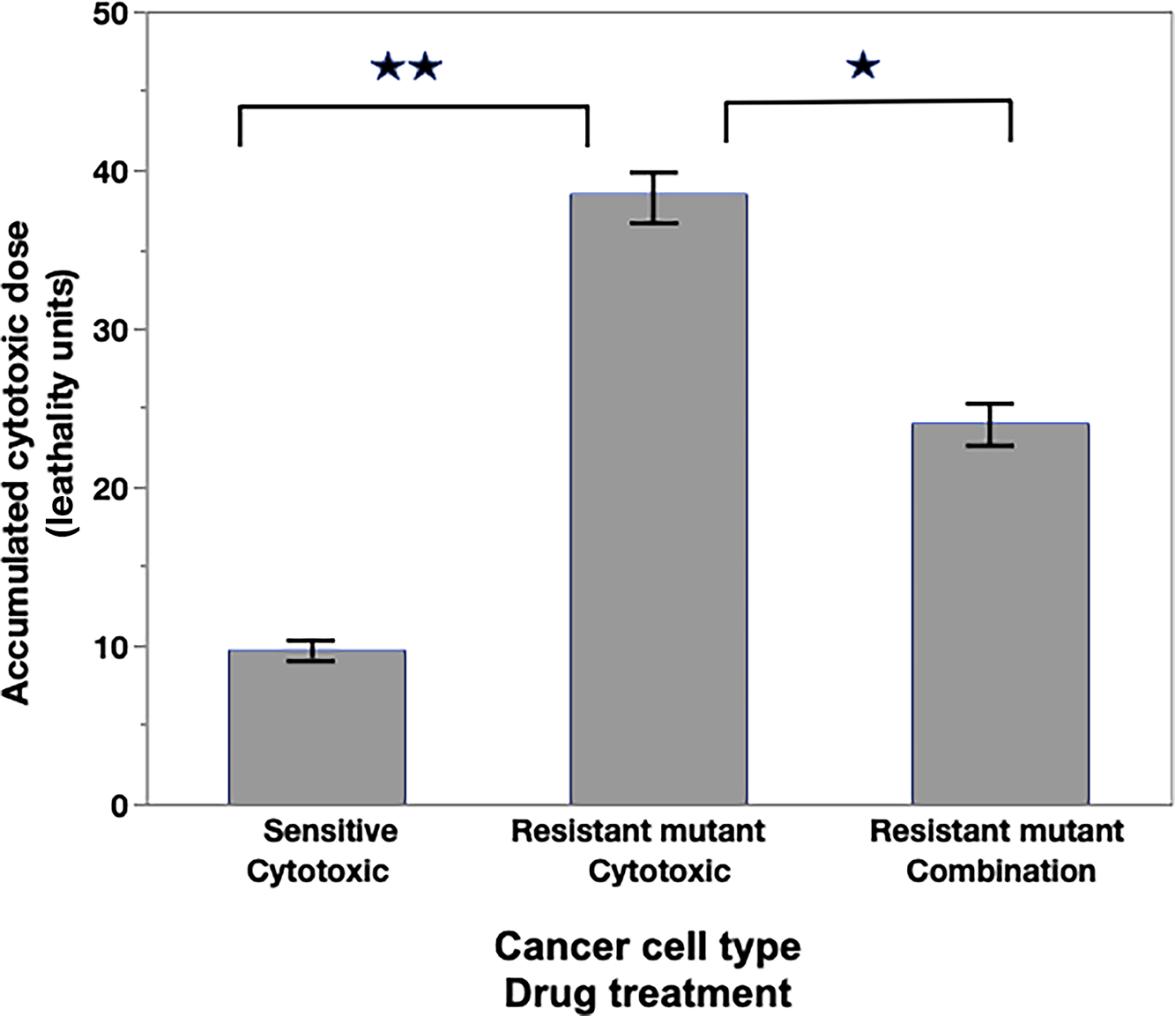
Accumulated cytotoxic dose for each cell type and each drug treatment. The sensitive cancer cell treated with a single cytotoxic drug is cured with an accumulated cytotoxic dose of 9.64 lethality units (left). The resistant mutant treated with a single cytotoxic drug accumulates more cytotoxic dose, 38.42 lethality units (middle). However, the resistant mutant treated with a combination of cytotoxic and apoptotic drugs accumulates less cytotoxic dose, 23.95 lethality units (right). Mean and SEM, *n* = 50, *t* test; *, *P* < 0.05; **, *P* < 0.01.

One strategy to eliminate relatively resistant cancer cells is to increase the cytotoxic dose. However, an increase in dose intensity is limited by an increase in undesirable side effects. The best increased dose schedule was the one that could cure 100% of the relatively resistant cancer cells (Table 1, line 2) with the least effect on crypt size, that is, that is 63.94% of the average size of an untreated crypt. This dose schedule required an increased in lethality from 5.75 to 8.25. Although the relatively resistant cancer cells could all be cured, the time to cure was increased from 71.01 to 194.56 steps, the equivalent to an increase of 11.8 to 33 days (Fig. 1, Cytotoxic, Resistant). However, the increase in time to cure resulted in an undesirable increase in the accumulated cytotoxic doses from 9.69 to 38.42 (Fig. 2, Resistant mutant, Cytotoxic).

Another strategy to eliminate cancer cells that are resistant to one drug is to treat the cells with a different drug with a different mechanism of action. For example, the drug sulindac induces apoptosis of cells at the lumen interface at the top of the crypt (4). A smaller number of cells per crypt was more efficient in removing cells, both normal cells and mutant cells. Sulindac reduces the number of cells per crypt whereas a cytotoxic drug interferes with DNA synthesis in dividing cells. Therefore, sulindac and 5-FU have different mechanisms of action.

Sulindac has been shown to be effective in the primary prevention of colon cancer in animal models (31, 50–53) and is a component of treatment of patients with familial adenomatous polyposis (54–56). Sulindac is being tested in chemoprevention clinical trials (57, 58). Although sulindac is an effective component in chemoprevention studies, it is not sufficient by itself to cure the slow-dividing cancer cells (Table 1, line 3). Slow-dividing cancer cells that are resistant to 5-FU are also resistant to sulindac. In this context, the slow dividing cancer cells are examples of multidrug-resistant cancer cells.

### An Effective Drug Combination Dose Schedule can be Identified that Eliminates Multidrug-resistant Cancer Cells with Least Harmful Side Effects

Another strategy to overcome the resistance of cancer cells to a single drug, or resistance to each of the two drugs separately, is to treat cancer cells with a combination of two drugs with different mechanisms of action (29, 30, 59). There are several challenges to combination chemotherapy with the intention of eliminating mutants that are resistant to each of the two drugs. The challenges include the following: achieving the highest percent cure of the cancer cells, having the least accumulated cytotoxic dose to reduce undesirable side effects, allowing normal cells in tissues to recover function, and determining time schedules that are practical to administer in the clinic. This is a multiobjective optimization problem that requires weighting several objectives against practical considerations (41).

In this example, two drugs with different mechanisms of action are the cytotoxic drug 5-FU and the apoptotic drug sulindac. To determine the dose schedule for each drug, a high-performance computer was used to simulate 170,000 different dose schedules for each of the two drugs in combination, noting that information from these 170,000 simulations was sufficient to predict the effects of dose schedules that were not simulated. The initial dose for each drug was administered simultaneously, and the time of subsequent doses of each drug was determined by its own interval. Of the complete set of dose schedules, 2,430 were found that cured all doubly resistant mutants in 100% of 50 independent trials and allowed crypts to recover (Supplementary Table S2).

However, most of these curative dose schedules are not practical to implement during the working hours of most infusion clinics. For instance, a dose schedule of four steps would require a patient to be in the infusion clinic for an inconvenient 16 hours, which is more than the usual 8 hours even 10 hours working day. A dose schedule with an interval between doses of 34 steps would require an interval of 5.7 days, which would quickly go out of synch with the 7-day week. Nevertheless, among the 2,430 drug combination dose schedules that could cure all double-resistant cancer cells, we identified a schedule that could be conveniently administered in the clinic (Table 1, line 4). This dose schedule has the cytotoxic drug 5-FU administered for 1 step (4 hours), every 42 steps (1 week), and has the apoptotic drug sulindac administered for 1 step (4 hours), every 84 steps (2 weeks).

For this convenient dose schedule, the average time to cure the doubly-resistant cancer cells by combination drug treatment was 133 steps (Table 1, line 4), equivalent to 24.17 days (Fig. 1, Combination/Resistant). This is a shorter time than the average time to cure the double-resistant cancer cells by 5-FU cytotoxic treatment alone, 194.56 steps (Table 1, line 2), equivalent to 32.43 days (Fig. 1, Cytotoxic/Resistant).

Although a shorter time to cure is desirable, another important objective is to reduce the accumulated cytotoxic doses to reduce the extent of undesirable side effects. The accumulated cytotoxic dose was calculated for each cancer cell type and each drug treatment. Each accumulated cytotoxic dose was calculated as lethality × (time to cure/interval). The selected combination drug treatment (Table 1, line 4) had a lower accumulated dose, 23.95 lethality units, to cure the resistant cancer cells than did the single drug (cytotoxic 5-FU) treatment, 38.42 lethality units (Fig. 2, Resistant mutant, Combination).

### Summary of Results

**FIGURE 3:**
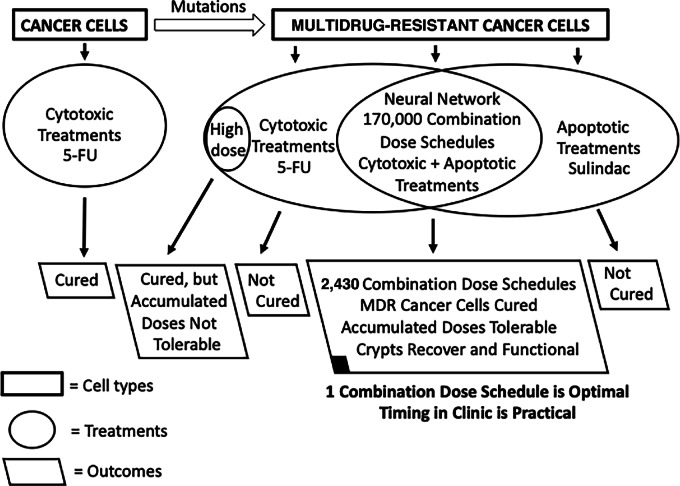
Flowchart summarizing the different outcomes when cancer cells or multidrug-resistant cancer cells are exposed to different treatments. Cancer cells could be eliminated (cured) by any one of 913 cytotoxic (5-FU) treatment dose schedules, out of 184,320 dose schedules simulated. Multidrug-resistant cancer cells could not be cured when exposed to any one of 184,320 cytotoxic (5-FU) treatment dose schedules, or separately to any one of 184,320 apoptotic (sulindac) treatment dose schedules; except by high-dose cytotoxic treatment when accumulated doses were not tolerable. An artificial neural network selected 170,000 combination cytotoxic and apoptotic dose schedules and among them discovered 2,430 that could cure all multidrug-resistant (MDR) cancer cells, had accumulated doses that are tolerable, and had crypts that recovered between doses and remained functional. One of these 2,430 dose schedules is optimal because it has timing of dose duration and intervals between doses that are practical to be implemented in the clinic as shown in Fig. 4.

The flowchart (Fig. 3) summarizes the different outcomes when cancer cells or multidrug-resistant cancer cells are exposed to different treatments. Cancer cells may be cured (eliminated) by any one of the 913 different cytotoxic (5-FU) treatments. Multidrug-resistant cancer cells cannot be cured by cytotoxic treatment alone, or by apoptotic (sulindac) treatment alone. Multidrug-resistant cancer cells may be cured by high doses of cytotoxic treatment alone; however, the required accumulated doses are nearly twice that of combination treatment, which may not be tolerated by the host. An artificial neural network generated 170,000 combinations of cytotoxic and apoptotic doses schedules. Of these, 2,430 can cure multidrug-resistant cancer cells, have accumulated dose schedules that are tolerable, have crypts that recover between doses and remain functional. Notably, one of these combination dose schedules is optimal because it has dose durations and intervals between doses that is practical to implement in the clinic.

**FIGURE 4:**
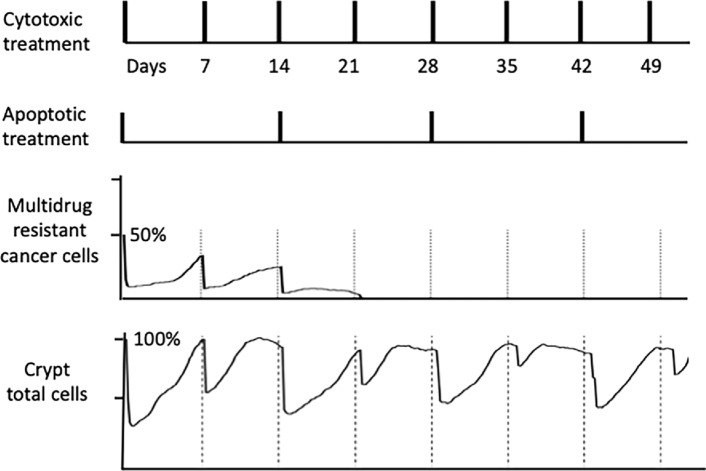
Timeline of curing multidrug-resistant cancer cells. Combination cytotoxic (5-FU) treatment (4 hours duration, interval 7 days) and apoptotic (sulindac) treatment (4 hours duration, interval 14 days) reduced the number of cancer cells from 50% of the number of cells in the colon crypt to zero in 22 days, while allowing the total number of crypt cells to recover between treatments. The crypt is cured of cancer cells and the crypts remain functional.

Figure 4 shows the results of the optimal combination chemotherapeutic dose schedule given in Table 1, line 4. A cytotoxic treatment of 4 hours duration at intervals of 7 days, and apoptotic treatment of 4 hours duration at intervals of 14 days, was applied to colon crypts containing 50% of multidrug-resistant cancer cells. The results showed that the number of cancer cells was reduced to zero in 22 days; the total number of normal crypt cells recovered between treatments and continued to function.

## Discussion

In this study, we have addressed four challenges associated with chemotherapy for early colon cancer. These include (i) eliminating all cancer cells while retaining normal crypt function, (ii) overcoming the evolution of cancer cells that may become multidrug resistant, (iii) determining dose schedules that have the least undesirable side effects, and (iv) developing dose schedules that have timing that is practical to administer in the clinic. This is a multiobjective optimization problem that requires weighting several objectives against practical considerations (41, 60).

Abnormally proliferating cells in the colon crypt may fill the crypt and form an adenoma, which is an early stage of colon cancer. If the crypt is treated with a cytotoxic drug such as 5-FU, the proliferation of the abnormal cells is inhibited, and an adenoma is not formed. Cytotoxic drugs will also inhibit the proliferation of normal cells in the crypt. However, stem cells, some of which are quiescent, are relatively resistant. Some intermittent dose schedules will eliminate proliferating cancer cells; however, between doses quiescent stem cells can become active, divide, and provide normal cells that proliferate and restore crypt function.

We have met the first challenge through the discovery of intermittent cytotoxic drug dose schedules that can remove cancer cells while retaining normal crypt function, with each dose schedule consisting of a set of values for three parameters; the duration of drug exposure, interval between drug exposure, and intensity of the drug dose. We solved this problem by utilizing an agent-based computer model of cell dynamics in the human colon crypt (33). This model was calibrated using measurements of crypts in human biopsy specimens, and its behavior was validated by reproducing experimental and clinical observations. The model allowed dimension reduction by providing constraints that limited all possible parameter sets to a realistic subset. A high-performance computer was used to generate a large number of parameter sets, each with three specific values representing drug duration, interval, and intensity. A subset of parameter values that eliminated cancer cells while retaining crypt function in all simulated trials was then identified.

We met the second challenge by determining dose schedules that eliminate cancer cells that are resistant to each of the two drugs, that is, multidrug resistance. For example, we simulated two drugs with different mechanisms of action. 5-FU inhibits DNA synthesis and is cytotoxic to dividing cells. Sulindac induces apoptosis of crypt cells at the lumen interface and reduces the size of the crypt making the crypt more efficient in removing cancer cells as they move up to the top of the crypt. The cytotoxic drug targets rapidly dividing cancer cells, whereas the apoptotic drug targets normal cells at the top of the crypt. The challenge of eliminating drug-resistant cancer cells was accomplished by identifying the schedules for the two drugs in combination. As described above, a large number of parameter sets of dose schedules were generated using a high-performance computer that simulated the removal of doubly resistant cancer cells from an agent-based model of colon crypts. Subsets of parameter sets that successfully eliminated cancer cells while retaining crypt function in all simulated trials were then identified.

We have met the third challenge by identifying a subset of combination drug dose schedules that can eliminate multidrug-resistant cancer cells with less undesirable side effects than a single cytotoxic drug treatment. This was accomplished by recognizing that some combination dose schedules would eliminate multidrug-resistant cancer cells in a shorter time and with less cytotoxic intensity, than the time and intensity required for single cytotoxic drug treatment. Such combination chemotherapy dose schedules accumulate less exposure to cytotoxic doses than a single cytotoxic therapy. Therefore, this combination chemotherapy would have less of an undesirable side effect than a single chemotherapy alone.

We have met the fourth challenge by recognizing that a specific dose schedule would have treatment times for each of the two drugs that are practical for implementation in the clinic. Most infusion clinics that administer chemotherapy drugs are open for only 8 to 10 hours a day, and only 5 or 6 days a week. This dose schedule includes two drugs administered for 4 hours at any time during the day. The cytotoxic drug 5-FU would be administered at intervals of one week, and the apoptotic drug sulindac administered at intervals of 2 weeks.

The dose schedule that we identified for combination chemotherapy (Table 1, line 4) has a specific dose duration, dose intervals, and dose intensities for each of the two drugs with different mechanisms of action. It can eliminate multidrug-resistant cancer cells with minimal undesirable side effects, and could be practical to administer in the clinic.

Several other agent-based models have been described and used in related studies. An agent-based model focused on stem cell dynamics in the colon crypt has been developed (61) and an agent-based model has been developed and colorectal tumor growth stimulated on a high-performance computer with the intention, in the future, of optimizing therapeutic strategies (62). A multiscale agent-based model has been used to investigate the synergistic effects of a large space of dose combinations using local and global optimization algorithms (63). Our own agent-based model differs from the other models in the following ways: it was calibrated with measurements of human biopsy specimens, and it reproduces the dynamics of colon crypt cells (stem cells, proliferating cells, and differentiated cells) that we observed experimentally (33).

The choice of dose schedules in combination drug therapy has been the subject of many investigations. For instance, a mathematical equation was derived to determine the time between maximally effective chemotherapy cycles (64). The optimal timing of drug sequences has been described as an important factor in multidrug adaptive therapies (65). The dosing frequencies and order of the two drugs were investigated using a stochastic model of tumor cell eradication that explored pharmacokinetics and drug combinations (66, 67). The question of sequential or simultaneous treatment of two drugs has been address in several studies. Bozic and colleagues (68) favor simultaneous, whereas Fang and colleagues (69) indicate that sequential inhibition minimizes toxicity while maintain efficacy, and Katouli and colleagues (70) conclude that “best-drug-first, worst-drug-longer” is best, even in the presence of cross-resistance. In our investigation, we avoided choosing between the order of the two drugs and the question of simultaneous or sequential. We start doses of two drugs at the same time and then, in a large number of different trials, each drug is applied with its own duration and interval. As the schedules progressed, the doses of each of the two drugs were being applied at different times. Thus, all schedule combinations, sequential and alternating, were evaluated.

An active line of investigation for combination chemotherapy is to utilize knowledge of the molecular biology of the drugs and their targets. This includes screening for the co-occurrence of altered genes in pharmacogenomics profiles (71), inhibiting multiple pathways instead of a single pathway (72), considering metabolic networks to predict combinations of synthetic lethal drugs (73), and coinhibition of several kinases. The high-throughput testing of drug combinations has been initiated in collaborative programs (74). Such screening is being enabled by machine-learning algorithms and mathematical models that evaluate the responses of multiple cell lines to multiple drugs that affect multiple targets (60, 75, 76). These efforts will provide information on new drug combinations. Our work complements these efforts by suggesting a method for determining the most efficacious dose schedules for new drug combinations.

This study has some limitations. As it focuses on drug-resistant cells in colon crypts during the early stages of colon cancer, it does not address other aspects of cancer progression such as metastasis. Moreover, it does not account for toxicities in other tissues and organs such as neuropathy, cardiotoxicity, and neutropenia. However, toxicity of the colon crypts is measurable as a loss of the total number of cells in the crypt; therefore, it may be considered as a quantitative surrogate for the toxicity of other tissues and organs. More comprehensive models are currently being developed (77–79).

Confidence in the predictions of these simulation results would be enhanced if they could be validated in a biological system. Mouse models are not appropriate because mouse colon crypts are smaller than human colon crypts and cell kinetics would be expected to be different. *In vitro* human colon stem cell–derived organoids and *in vivo* patient-derived xenografts would not be appropriate because the human crypts would not be in the real human microenvironment and human physiologic environment.

A clinical trial could provide the necessary information to test our predictions. It would not be necessary to design a new clinical trial specifically to test the predictions. Rather, it would be possible to take advantage of clinical trials designed for other purposes. For instance, clinical trials designed to test the efficacy of new candidate drugs and new combinations of drugs are common. Such trials would be informative if they included each of several features: patients with multidrug-resistant colon cancer treated with a candidate cytotoxic drug in combination with sulindac and several different dose schedules. The results of such trials could be used to test our prediction that a cytotoxic drug and sulindac at intervals of 7 and 14 days, respectively, would have a greater 3-year survival rate or a longer median progression-free survival time than other dose schedules. However, a recent search of the website ClinicalTrials.gov does not find a trial with each of these features. A future trial may be informative.

In summary, we have described a method to determine dose schedules in combination chemotherapy that eliminates colon cancer cells that have evolved resistance to each of two drugs, retain normal colon crypt function, has minimal side effects, and would be practical to administer (Fig. 4). This was accomplished by generating thousands of dose schedules with a high-performance computer that simulated cell dynamics in an agent-based model of colon crypts, and identifying the subset of dose schedules that are the most effective.

## Supplementary Material

Table S1 The 913 different dose schedules that cured all cancer cells in 100% of 50 simulation runs while retaining crypt function.

Table S2 The 2430 dose schedules that cured all doubly resistant mutants in 100% of 50 independent trials and allowed crypts to recover.
